# Who Let the Dogs Out? a Case of Delirium Induced by Lyme Borreliosis in a Patient With a Severe Intellectual Development Disorder

**DOI:** 10.1192/bjo.2022.362

**Published:** 2022-06-20

**Authors:** Joana Marques Pinto, José Diogo Andrade, Joana Andrade, Vera Martins

**Affiliations:** CHUC, Coimbra, Portugal

## Abstract

**Aims:**

Lyme borreliosis is caused by certain genospecies of the Borrelia burgdorferi sensu lato complex, which are transmitted by hard ticks of the genus Ixode. The most common clinical manifestation is erythema migrans, an expanding skin redness that usually develops at the site of a tick bite and eventually resolves regardless of antibiotic treatment. It may result in a range of clinical manifestations involving different organ systems, and can lead to persistent sequelae in a subset of cases.

**Methods:**

We describe a case of a 47-year-old male, with severe intellectual development disorder (IDD), who presented with behavioural changes, aggressiveness, psychomotor agitation and confusion. 15 days prior to admission in the psychiatry ward he had recurred several times to the emergency department with similar clinical presentation, and had been discharged following adjustments to his medication. After showing no improvement and no response to treatment he was admitted. He then presented fever and laboratory study (LS) revealed increased inflammatory markers. His family also informed he came from a rural area and had contact with wild dogs. No tick bite or erythema was identified during physical examination. Nevertheless a serologic study for Borrelia burgdorferi was performed and turned out positive. An antibiotic regimen was administered and and the patient's symptoms fully remitted 48 hours after treatment was initiated.

**Results:**

Borreliosis usually presents erythema at the site of the tick bite which could have already resolved when the patient was examined. It was first assumed that the clinical manifestations were part of his psychiatric condition. An infectious etiology was presumed after the onset of fever and increased inflammatory markers were identified. Given the patient's context, Borrelia in particular was considered a likely hypothesis. This case illustrates the difficulties of differential diagnosis inherent to patients with IDD, both because of the pathology itself, which can mask such clinical manifestations as delirium, and of the stigma associated with mental health patients, which frequently cuts the diagnostic work-up of organic causes short.

**Conclusion:**

This case highlights the clinical challenge patients with IDD represent. Differential diagnosis can be elusive, especially in the context of infectious diseases like borrelia, as they can present with unspecific clinical manifestations in this subgroup of patients, and hence why a complete and thorough clinical evaluation is essential. This case also illustrates that mental health patients suffer from stigma: Being branded a “psychiatric patient” created a 16-day delay between onset of symptoms and appropriate treatment initiation- antibiotics.

